# The Road Not Taken: Endoscopic Management of an Unexpected Enteric Biliary Fistula

**DOI:** 10.7759/cureus.85636

**Published:** 2025-06-09

**Authors:** Raahi Patel, Varshita Goduguchinta, Shil Punatar, Zohair Ahmed

**Affiliations:** 1 Internal Medicine, Franciscan Health, Olympia Fields, USA; 2 Gastroenterology, Franciscan Health, Olympia Fields, USA

**Keywords:** ascending cholangitis, choledocoduodenal fistula, ercp, laparoscopic cholecystectomy, sepsis

## Abstract

Ascending cholangitis represents an endoscopic emergency demanding prompt recognition and intervention, typically via endoscopic retrograde cholangiopancreatography (ERCP). While routine ERCP access is achieved through the major papilla in the duodenum, this case highlights a unique anatomical variation: a choledochoduodenal fistulous tract in an 81-year-old male presenting with ascending cholangitis. Notably, the ERCP was successfully performed by utilizing this fistulous tract as the primary access point for intervention. This case underscores the importance of recognizing such rare anatomical anomalies in biliary diseases and their implications for endoscopic management.

## Introduction

Ascending cholangitis is an infection of the biliary tree characterized by fever, jaundice, and abdominal pain, most commonly due to biliary obstruction [1]. Prompt initiation of IV fluids and antibiotics is vital in these patients. Current mortality benefits stem from this early recognition and intervention with advancements in endoscopic retrograde cholangiopancreatography (ERCP) to allow for sphincterotomy and stone extraction/biliary drainage [2]. Early recognition aims to prevent escalation to a more severe form called acute obstructive suppurative cholangitis (AOSC). With this case, we highlight unique and lesser-documented anatomical variations and a subsequent approach to endoscopic treatment of ascending cholangitis.

This article was previously presented as an abstract at the 2024 American College of Gastroenterology Annual Meeting on October 28, 2024, in Philadelphia, PA.

## Case presentation

An 81-year-old Caucasian male with past medical history notable for elevated bilirubin for the past two years, recent cholecystectomy, persistent atrial fibrillation on rivaroxaban, moderate aortic regurgitation, heart failure with recovered ejection fraction, hypertension, and hyperlipidemia presented with complaints of shortness of breath and epigastric abdominal pain for several days. Aggravating factors included exacerbation of pain upon inspiration; however, he denied any association between his epigastric pain with food intake.

Laboratory evaluation upon admission was notable for alkaline phosphatase of 221, aspartate aminotransferase (AST) of 252, alanine aminotransferase (ALT) of 120, and total bilirubin of 3.0. Lactic acid was also elevated at 3.2, and leukocytosis was 11.58 (Table 1). CT chest/abdomen/pelvis revealed pulmonary emphysema but was otherwise unremarkable. The gastroenterology team was consulted, and given the chronicity of the patient's elevation in liver enzymes, a plan was made to pursue a chronic liver disease evaluation with hepatitis serologies, anti-smooth muscle antibody, anti-nuclear antibody (ANA), and iron studies, along with right upper quadrant ultrasound. Shortly after, there was a change in the patient’s clinical status for which a rapid response was called for hypotension and bradycardia. Presentation then was notable for the new onset of jaundice; complaints of chest discomfort, fever, nausea, and vomiting; and worsened abdominal tenderness on palpation. Fluid resuscitation and broad-spectrum antibiotics were empirically initiated.

**Table 1:** Patient's lab values on admission

Lab Test	Patient's Value	Normal Range	Units	Notes/Interpretation
ALP (Alkaline Phosphatase)	221	30-120	U/L	Elevated
AST (Aspartate Aminotransferase)	252	10-40	U/L	Significantly Elevated
ALT (Alanine Aminotransferase)	120	7-55	U/L	Elevated
Total Bilirubin	3	0.3-1.0	mg/dL	Elevated
Lactic Acid	3.2	0.5-2.2	mmol/L	Elevated
WBC (White Blood Cell Count)	11.58	4.0-10.0	x 10^9^/L	Slightly Elevated

**Figure 1:**
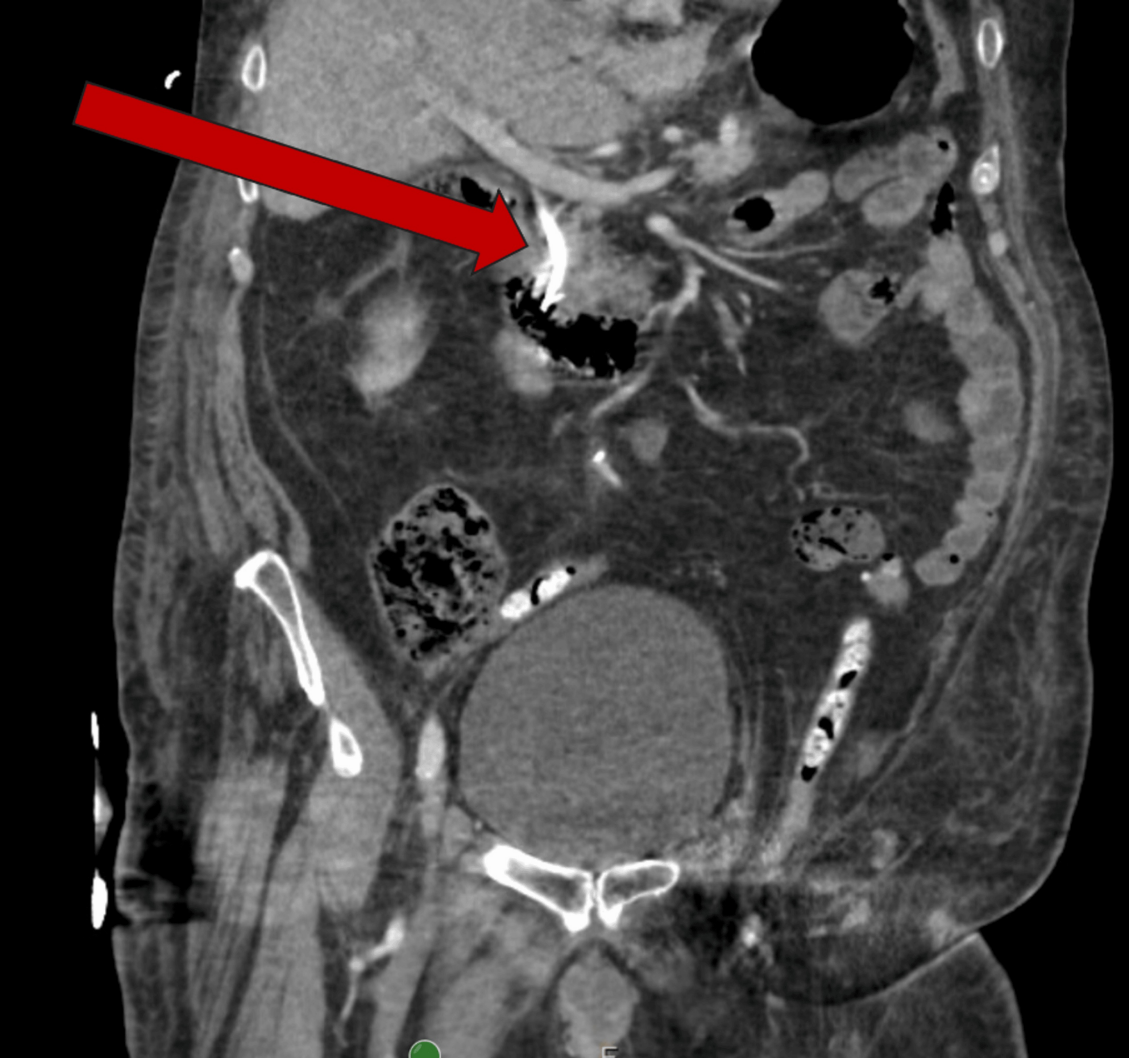
CT abdomen & pelvis showing biliary stent placement through a fistulous tract between the duodenum and common bile duct, highlighting a choledochoduodenal fistula

Given high suspicion for biliary tract as the nidus of infection, a stat right upper quadrant (RUQ) ultrasound was performed and showed surgically absent gallbladder with expected dilated common bile duct and several foci of pneumobilia likely related to cholecystectomy. The decision was made to pursue an emergent ERCP. During the ERCP, a fistulous tract was found proximal to the papilla, which was utilized to conduct the procedure. Through this tract, a diverticulum was found that allowed access to the common bile duct. This common bile duct was cannulated, a balloon sweep was performed, and copious amounts of pus, sludge, and two stones were removed via the fistulous tract. A plastic biliary stent was placed (Figure 1, arrowhead). No sphincterotomy was performed, given that the procedure was conducted via a fistula and ongoing anticoagulant use. The patient progressed well throughout the hospital course and was discharged in stable condition.

## Discussion

AOSC is a severe form of ascending cholangitis seen when pus collection is evident in the biliary tree. ERCP is the gold standard for choledocholithiasis, along with AOSC, with early recognition being vital due to the high risk of sepsis and mortality rates [3]. According to a systematic review and meta-analysis by Du et al., early ERCP was associated with reduced mortality at various hourly timelines, including <24, <48, and <72 hours. Specifically, those who received urgent ERCP <24 hours of symptom presentation vs. those >24 hours had a 20% decrease in in-hospital mortality [4]. ERCP was urgently conducted in the case mentioned above but was met with an atypical anatomic finding.

**Figure 2:**
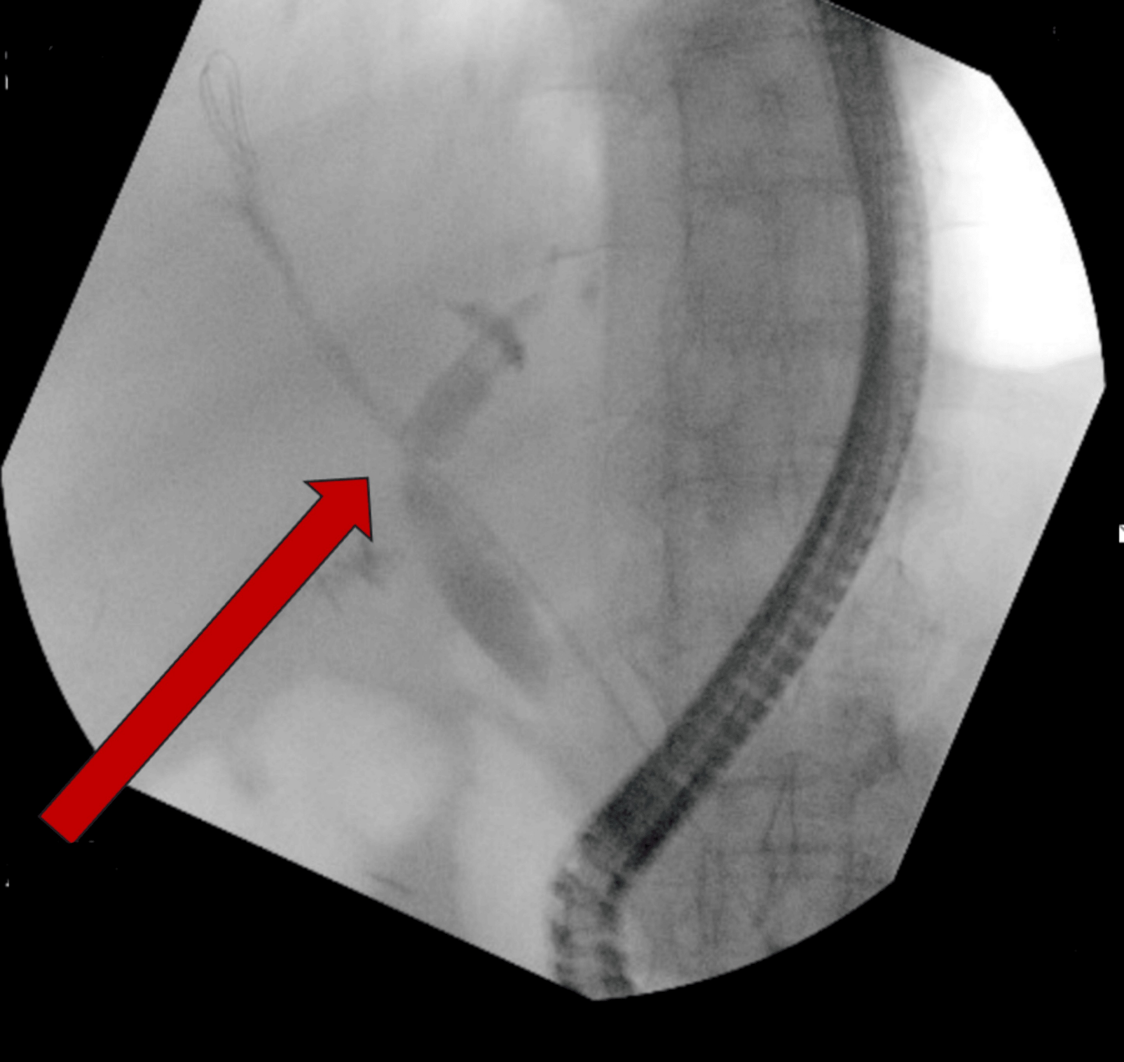
Imaging during ERCP highlighting prior cholecystectomy ERCP: acute obstructive suppurative cholangitis

In our case, we aim to highlight the fistulous communication identified between the common bile duct and the duodenum, consistent with a choledochoduodenal fistula, which ultimately aided in the drainage of pus, sludge, and biliary stones during the ERCP. The formation of the fistula may be secondary to multifactorial causes. Previous cholecystectomy (Figure 2) is a known risk factor for bile duct injury, with studies demonstrating an incidence of around 0.4-0.6% in subsequent biliary procedures [5]. Additionally, the formation of a fistula from iatrogenic injuries occurring during cholecystectomy is about 0.3-0.6% [6]. It is possible that the patient's cholecystectomy played a role in the formation of the fistula identified during ERCP. In contrast to surgically induced choledochoduodenal fistulas, spontaneous fistulas tend to be less prevalent. Etiologies of these non-iatrogenic fistulas often involve the presence of gallstones seen in cholelithiasis or choledocholithiasis [7]. The chronicity of cholelithiasis is suspected, potentially contributing to the atypical presentation observed [8]. A study conducted by Jorge et al. highlighted that, of the 14 patients who were found with a choledochoduodenal fistula via ERCP, 12 were due to iatrogenic injuries, while only two resulted spontaneously [9]. In another study by Wang et al., a 65-year-old woman was found to have a proximal choledochoduodenal fistula as a result of prolonged, untreated choledocholithiasis [10].

## Conclusions

This case vividly illustrates that anatomical variations, such as a choledochoduodenal fistulous tract, can present unique challenges and opportunities in the management of acute biliary emergencies. Despite the atypical access point, successful ERCP was performed, leading to effective drainage and clinical improvement in a patient with ascending cholangitis. This report underscores the critical importance for endoscopists to maintain a high index of suspicion for anatomical anomalies, particularly in patients with complex biliary histories, as recognizing and adapting to these variations can be pivotal for successful intervention and optimal patient outcomes. We hope to highlight the considerations of fistula formation as sequelae to cholecystectomy, adding the notions of anatomical variants in cholangitis, and discussing alternative methods of sphincterotomy with stent placement in medical literature and discussions on the completion of ERCP procedures.
